# Aqua­chlorido{μ-6,6′-dieth­oxy-2,2′-[1,2-phenyl­enebis(nitrilo­methyl­idyne)]diphenolato}copper(II)sodium(I) *N*,*N*-dimethyl­formamide solvate

**DOI:** 10.1107/S1600536809051150

**Published:** 2009-12-12

**Authors:** Xiao-Jian Ma

**Affiliations:** a School of Chemistry & Chemical Technology, Shandong University, Jinan 250100, People’s Republic of China

## Abstract

In the heterometallic dinuclear title compound, [CuNa(C_24_H_22_N_2_O_4_)Cl(H_2_O)]·C_3_H_7_NO, the Cu^II^ ion is coord­inated in a square-planar geometry by two N atoms and two O atoms of the 6,6′-dieth­oxy-2,2′-[1,2-phenyl­enebis(nitrilo­methyl­­idyne)]diphenolate ligand. The Na^I^ ion is hexa­coordinated by four O atoms of the ligand, defining the equatorial plan, and by one O atom of the water mol­ecule and one Cl atom occuping axial positions. The Cu^II^ and Na^I^ ions are bridged by two phenolate O atoms.

## Related literature

For related heteronuclear complexes, see: Karlin (1993 ▶); Ni *et al.* (2005 ▶). For related structures, see: Bian (2008 ▶); Xiao & Zhu (2003 ▶). For the synthesis of 6,6′-dieth­yloxy-2,2′-[1,2-phenyl­enebis(nitrilo­methyl­idyne)]diphenol and its Cu complex, see: Lo *et al.* (2004 ▶); Sui *et al.* (2007 ▶).
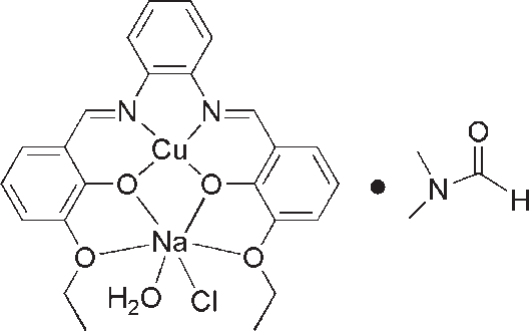


## Experimental

### 

#### Crystal data

[CuNa(C_24_H_22_N_2_O_4_)Cl(H_2_O)]·C_3_H_7_NO
*M* *_r_* = 615.53
Monoclinic, 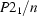

*a* = 12.2528 (17) Å
*b* = 19.566 (3) Å
*c* = 12.4901 (17) Å
β = 111.653 (2)°
*V* = 2783.1 (7) Å^3^
*Z* = 4
Mo *K*α radiation
μ = 0.94 mm^−1^
*T* = 298 K
0.15 × 0.10 × 0.08 mm

#### Data collection

Bruker APEXII CCD area-detector diffractometer
Absorption correction: multi-scan (*SADABS*; Sheldrick, 2003 ▶) *T*_min_ = 0.872, *T*_max_ = 0.928
13672 measured reflections
4903 independent reflections
4233 reflections with *I* > 2σ(*I*)
*R*_int_ = 0.024

#### Refinement

*R*[*F* ^2^ > 2σ(*F* ^2^)] = 0.032
*wR*(*F* ^2^) = 0.092
*S* = 1.07
4903 reflections
354 parameters
H-atom parameters constrained
Δρ_max_ = 0.51 e Å^−3^
Δρ_min_ = −0.46 e Å^−3^

### 

Data collection: *APEX2* (Bruker, 2004 ▶); cell refinement: *SAINT-Plus* (Bruker, 2001 ▶); data reduction: *SAINT-Plus*; program(s) used to solve structure: *SHELXS97* (Sheldrick, 2008 ▶); program(s) used to refine structure: *SHELXL97* (Sheldrick, 2008 ▶); molecular graphics: *SHELXTL* (Sheldrick, 2008 ▶); software used to prepare material for publication: *SHELXL97*.

## Supplementary Material

Crystal structure: contains datablocks global, I. DOI: 10.1107/S1600536809051150/is2489sup1.cif

Structure factors: contains datablocks I. DOI: 10.1107/S1600536809051150/is2489Isup2.hkl

Additional supplementary materials:  crystallographic information; 3D view; checkCIF report

## Figures and Tables

**Fig. 1.**
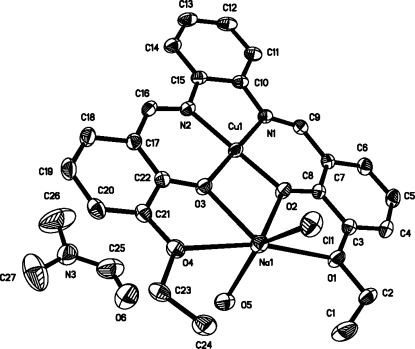
A view of the title compound with the atom-labelling scheme. Displacement ellipsoids are drawn at the 30% probability level.

## supplementary crystallographic information

### Comment

Heterometallic complexes have been intensively studied owing to their unique physical and chemical properties (Ni *et al.*, 2005). In addition, these compounds exist at the active sites of many metalloenzymes and play important roles in biological systems (Karlin, 1993). Therefore, investigation of the synthesis and the crystal structures of these heterometallic compounds is necessary in order to further widening the application of the compounds. Herein, a novel heterometallic nuclear (Cu^II^Na^I^) compound has been obtained by step-by-step method and its structure is depicted.

As shown in Fig.1, the compound I is a dinuclear neutral complex with a planar square configuration. The Cu(II) atom is coordinated in a planar square geometry with the basal square formed by two nitrogen atoms and two oxygen atoms from the 6,6'-diethyloxy-2,2'-[1,2-phenylenebis(nitrilomethylidyne)]diphenolate (*L*) ligand. The Na(I) atom is coordinated by four oxygen atoms from the ligand, one oxygen atom from water and one chlorine atom. The bond lengths of Cu—O, Cu—N and Na—Cl are normal (Xiao *et al.*, 2003).

### Experimental

The H_2_L ligand and complex CuL was synthesized according to the previous literature (Lo *et al.*, 2004; Sui *et al.* 2007). The compound I was obtained by allowing the mixure of CuL (0.047 g, 0.1 mmol) and NaCl (0.006 g, 0.1 mmol) being stirred in the DMF solution at room temperature for 1 h, then filtered, suitable brown crystals were obtained *via* slow evaporation of the filtrate at room temperature (yield: about 45%)

### Refinement

All H-atoms bound to the C atoms were refined using a riding model, with C—H = 0.93 Å and *U*_iso_(H) = 1.2*U*_eq_(C) for aromatic atoms, C—H = 0.97 Å and *U*_iso_(H) = 1.2*U*_eq_(C) for methylene atoms, and C—H = 0.96 Å and *U*_iso_(H) = 1.5*U*_eq_(C) for methyl atoms. The H atoms of the water molecule were contrained, with O—H = 0.85 Å, and with *U*_iso_(H) = 1.5*U*_eq_(O).

### Crystal data

**Table:**

[CuNa(C_24_H_22_N_2_O_4_)Cl(H_2_O)]·C_3_H_7_NO	*F*(000) = 1276
*M**_r_* = 615.53	*D*_x_ = 1.469 Mg m^−^^3^
Monoclinic, *P*2_1_/*n*	Mo *K*α radiation, λ = 0.71073 Å
Hall symbol: -P 2yn	Cell parameters from 7372 reflections
*a* = 12.2528 (17) Å	θ = 2.3–27.5°
*b* = 19.566 (3) Å	µ = 0.94 mm^−^^1^
*c* = 12.4901 (17) Å	*T* = 298 K
β = 111.653 (2)°	Needle, brown
*V* = 2783.1 (7) Å^3^	0.15 × 0.10 × 0.08 mm
*Z* = 4	

### Data collection

**Table:**

Bruker APEXII CCD area-detector diffractometer	4903 independent reflections
Radiation source: fine-focus sealed tube	4233 reflections with *I* > 2σ(*I*)
graphite	*R*_int_ = 0.024
Detector resolution: 0 pixels mm^-1^	θ_max_ = 25.0°, θ_min_ = 2.0°
φ and ω scans	*h* = −14→14
Absorption correction: multi-scan (*SADABS*; Sheldrick, 2003)	*k* = −23→23
*T*_min_ = 0.872, *T*_max_ = 0.928	*l* = −12→14
13672 measured reflections	

### Refinement

**Table:**

Refinement on *F*^2^	Primary atom site location: structure-invariant direct methods
Least-squares matrix: full	Secondary atom site location: difference Fourier map
*R*[*F*^2^ > 2σ(*F*^2^)] = 0.032	Hydrogen site location: inferred from neighbouring sites
*wR*(*F*^2^) = 0.092	H-atom parameters constrained
*S* = 1.07	*w* = 1/[σ^2^(*F*_o_^2^) + (0.055*P*)^2^ + 0.6422*P*] where *P* = (*F*_o_^2^ + 2*F*_c_^2^)/3
4903 reflections	(Δ/σ)_max_ < 0.001
354 parameters	Δρ_max_ = 0.51 e Å^−^^3^
0 restraints	Δρ_min_ = −0.46 e Å^−^^3^

### Special details

**Table:**

Geometry. All e.s.d.'s (except the e.s.d. in the dihedral angle between two l.s. planes) are estimated using the full covariance matrix. The cell e.s.d.'s are taken into account individually in the estimation of e.s.d.'s in distances, angles and torsion angles; correlations between e.s.d.'s in cell parameters are only used when they are defined by crystal symmetry. An approximate (isotropic) treatment of cell e.s.d.'s is used for estimating e.s.d.'s involving l.s. planes.
Refinement. Refinement of *F*^2^ against ALL reflections. The weighted *R*-factor *wR* and goodness of fit *S* are based on *F*^2^, conventional *R*-factors *R* are based on *F*, with *F* set to zero for negative *F*^2^. The threshold expression of *F*^2^ > σ(*F*^2^) is used only for calculating *R*-factors(gt) *etc*. and is not relevant to the choice of reflections for refinement. *R*-factors based on *F*^2^ are statistically about twice as large as those based on *F*, and *R*- factors based on ALL data will be even larger.

### Fractional atomic coordinates and isotropic or equivalent isotropic displacement parameters (Å^2^)

**Table:**

	*x*	*y*	*z*	*U*_iso_*/*U*_eq_	
C1	0.4298 (3)	0.29377 (13)	0.0350 (3)	0.0753 (8)	
H1A	0.4592	0.3390	0.0331	0.113*	
H1B	0.4629	0.2761	0.1121	0.113*	
H1C	0.3458	0.2952	0.0108	0.113*	
C2	0.4633 (2)	0.24849 (10)	−0.0442 (2)	0.0472 (5)	
H2A	0.4304	0.2659	−0.1224	0.057*	
H2B	0.5480	0.2467	−0.0207	0.057*	
C3	0.44513 (16)	0.13019 (10)	−0.09867 (16)	0.0350 (4)	
C4	0.51898 (18)	0.13645 (11)	−0.15763 (17)	0.0426 (5)	
H4	0.5511	0.1789	−0.1626	0.051*	
C5	0.5466 (2)	0.07961 (12)	−0.21056 (19)	0.0488 (5)	
H5	0.5972	0.0843	−0.2502	0.059*	
C6	0.4997 (2)	0.01743 (11)	−0.20428 (19)	0.0439 (5)	
H6	0.5205	−0.0204	−0.2377	0.053*	
C7	0.41968 (17)	0.00950 (10)	−0.14769 (17)	0.0337 (4)	
C8	0.39040 (16)	0.06668 (9)	−0.09376 (15)	0.0314 (4)	
C9	0.37331 (17)	−0.05756 (10)	−0.14566 (16)	0.0352 (4)	
H9	0.4006	−0.0926	−0.1795	0.042*	
C10	0.25323 (16)	−0.14097 (10)	−0.10326 (16)	0.0356 (4)	
C11	0.27395 (18)	−0.19532 (10)	−0.16551 (18)	0.0430 (5)	
H11	0.3185	−0.1886	−0.2109	0.052*	
C12	0.2284 (2)	−0.25866 (11)	−0.1596 (2)	0.0508 (6)	
H12	0.2427	−0.2949	−0.2007	0.061*	
C13	0.1616 (2)	−0.26903 (11)	−0.0931 (2)	0.0522 (6)	
H13	0.1323	−0.3124	−0.0891	0.063*	
C14	0.13774 (19)	−0.21583 (11)	−0.03232 (18)	0.0459 (5)	
H14	0.0917	−0.2232	0.0114	0.055*	
C15	0.18307 (17)	−0.15112 (10)	−0.03704 (16)	0.0363 (4)	
C16	0.09870 (17)	−0.09228 (10)	0.08093 (17)	0.0380 (4)	
H16	0.0617	−0.1331	0.0855	0.046*	
C17	0.07786 (17)	−0.03460 (11)	0.14138 (17)	0.0385 (4)	
C18	0.00260 (19)	−0.04354 (13)	0.20363 (18)	0.0487 (5)	
H18	−0.0308	−0.0861	0.2043	0.058*	
C19	−0.0213 (2)	0.00910 (13)	0.2621 (2)	0.0526 (6)	
H19	−0.0707	0.0021	0.3025	0.063*	
C20	0.02749 (19)	0.07394 (13)	0.26241 (18)	0.0483 (5)	
H20	0.0110	0.1095	0.3035	0.058*	
C21	0.09936 (17)	0.08496 (11)	0.20221 (16)	0.0387 (4)	
C22	0.12742 (16)	0.03082 (10)	0.14010 (16)	0.0351 (4)	
C23	0.1425 (2)	0.20234 (11)	0.26047 (19)	0.0492 (5)	
H23A	0.1826	0.1917	0.3414	0.059*	
H23B	0.0612	0.2130	0.2472	0.059*	
C24	0.2003 (2)	0.26168 (12)	0.2272 (2)	0.0617 (7)	
H24A	0.1974	0.3009	0.2723	0.092*	
H24B	0.1598	0.2717	0.1469	0.092*	
H24C	0.2806	0.2505	0.2409	0.092*	
C25	0.3559 (3)	0.03387 (18)	0.4305 (3)	0.0904 (11)	
H25	0.3942	0.0080	0.3925	0.108*	
C26	0.3037 (6)	−0.0754 (2)	0.4803 (5)	0.169 (2)	
H26A	0.2303	−0.0927	0.4274	0.253*	
H26B	0.3190	−0.0942	0.5554	0.253*	
H26C	0.3657	−0.0882	0.4546	0.253*	
C27	0.2338 (4)	0.0311 (3)	0.5408 (3)	0.139 (2)	
H27A	0.2853	0.0446	0.6165	0.208*	
H27B	0.1747	0.0008	0.5468	0.208*	
H27C	0.1970	0.0709	0.4975	0.208*	
N1	0.29705 (13)	−0.07355 (8)	−0.10098 (13)	0.0328 (3)	
N2	0.16488 (13)	−0.09243 (8)	0.02039 (13)	0.0346 (4)	
N3	0.2978 (2)	−0.00202 (13)	0.4854 (2)	0.0744 (7)	
O1	0.41778 (13)	0.18169 (7)	−0.03841 (13)	0.0450 (3)	
O2	0.31963 (12)	0.06507 (7)	−0.03795 (12)	0.0401 (3)	
O3	0.19590 (12)	0.04606 (7)	0.08560 (12)	0.0410 (3)	
O4	0.14894 (13)	0.14563 (7)	0.19102 (12)	0.0466 (4)	
O5	0.43276 (14)	0.15357 (8)	0.24781 (14)	0.0584 (4)	
H5A	0.4787	0.1878	0.2615	0.088*	
H5B	0.4225	0.1437	0.3097	0.088*	
O6	0.3640 (2)	0.09160 (14)	0.4248 (2)	0.1041 (8)	
Cu1	0.243871 (19)	−0.012818 (12)	−0.008330 (19)	0.03398 (10)	
Na1	0.27089 (7)	0.15365 (4)	0.06022 (7)	0.0437 (2)	
Cl1	0.10362 (6)	0.23100 (3)	−0.10680 (6)	0.06409 (19)	

### Atomic displacement parameters (Å^2^)

**Table:**

	*U*^11^	*U*^22^	*U*^33^	*U*^12^	*U*^13^	*U*^23^
C1	0.084 (2)	0.0393 (14)	0.112 (2)	−0.0106 (13)	0.0475 (18)	−0.0153 (14)
C2	0.0468 (12)	0.0323 (11)	0.0554 (13)	−0.0061 (9)	0.0103 (10)	0.0059 (9)
C3	0.0335 (10)	0.0338 (10)	0.0367 (10)	0.0046 (8)	0.0116 (8)	0.0060 (8)
C4	0.0426 (11)	0.0420 (11)	0.0472 (11)	−0.0018 (9)	0.0211 (9)	0.0092 (9)
C5	0.0515 (13)	0.0535 (14)	0.0535 (13)	−0.0004 (11)	0.0335 (11)	0.0037 (10)
C6	0.0482 (12)	0.0449 (12)	0.0468 (12)	0.0051 (10)	0.0273 (10)	−0.0013 (9)
C7	0.0338 (10)	0.0343 (10)	0.0327 (10)	0.0048 (8)	0.0119 (8)	0.0035 (8)
C8	0.0301 (9)	0.0321 (10)	0.0327 (9)	0.0031 (8)	0.0125 (8)	0.0043 (8)
C9	0.0373 (10)	0.0332 (10)	0.0346 (10)	0.0057 (8)	0.0126 (8)	−0.0010 (8)
C10	0.0321 (10)	0.0312 (10)	0.0365 (10)	0.0016 (8)	0.0046 (8)	−0.0003 (8)
C11	0.0410 (11)	0.0382 (11)	0.0438 (11)	0.0034 (9)	0.0088 (9)	−0.0067 (9)
C12	0.0501 (13)	0.0339 (11)	0.0555 (13)	0.0027 (10)	0.0042 (11)	−0.0101 (10)
C13	0.0553 (14)	0.0308 (11)	0.0575 (14)	−0.0068 (10)	0.0056 (11)	−0.0018 (10)
C14	0.0453 (12)	0.0382 (12)	0.0480 (12)	−0.0072 (9)	0.0100 (10)	0.0025 (9)
C15	0.0347 (10)	0.0307 (10)	0.0365 (10)	−0.0019 (8)	0.0049 (8)	0.0011 (8)
C16	0.0333 (10)	0.0369 (11)	0.0423 (11)	−0.0063 (8)	0.0121 (9)	0.0054 (8)
C17	0.0318 (10)	0.0471 (12)	0.0360 (10)	−0.0022 (9)	0.0117 (8)	0.0031 (9)
C18	0.0420 (12)	0.0627 (15)	0.0455 (12)	−0.0087 (11)	0.0208 (10)	0.0049 (11)
C19	0.0457 (13)	0.0757 (17)	0.0469 (13)	−0.0026 (12)	0.0295 (11)	0.0030 (11)
C20	0.0421 (12)	0.0668 (15)	0.0392 (11)	0.0066 (11)	0.0188 (9)	−0.0046 (10)
C21	0.0335 (10)	0.0485 (12)	0.0345 (10)	0.0044 (9)	0.0129 (8)	0.0000 (9)
C22	0.0306 (10)	0.0414 (11)	0.0327 (10)	0.0015 (8)	0.0107 (8)	0.0013 (8)
C23	0.0512 (13)	0.0496 (13)	0.0485 (12)	0.0094 (10)	0.0202 (10)	−0.0140 (10)
C24	0.0727 (17)	0.0460 (14)	0.0739 (16)	0.0052 (12)	0.0359 (14)	−0.0157 (12)
C25	0.100 (3)	0.063 (2)	0.086 (2)	−0.0051 (19)	0.010 (2)	−0.0020 (17)
C26	0.197 (5)	0.077 (3)	0.176 (5)	−0.047 (3)	0.004 (4)	0.027 (3)
C27	0.088 (3)	0.255 (6)	0.069 (2)	0.035 (4)	0.024 (2)	0.023 (3)
N1	0.0344 (8)	0.0278 (8)	0.0354 (8)	0.0014 (6)	0.0119 (7)	−0.0012 (6)
N2	0.0334 (8)	0.0309 (8)	0.0380 (9)	−0.0028 (7)	0.0114 (7)	0.0009 (7)
N3	0.0758 (17)	0.0785 (17)	0.0646 (15)	−0.0128 (13)	0.0209 (13)	0.0074 (12)
O1	0.0520 (9)	0.0299 (7)	0.0622 (9)	−0.0036 (6)	0.0318 (7)	−0.0011 (6)
O2	0.0459 (8)	0.0293 (7)	0.0566 (8)	−0.0021 (6)	0.0323 (7)	−0.0029 (6)
O3	0.0459 (8)	0.0350 (7)	0.0535 (8)	−0.0052 (6)	0.0316 (7)	−0.0077 (6)
O4	0.0569 (9)	0.0404 (8)	0.0520 (9)	0.0023 (7)	0.0311 (7)	−0.0096 (6)
O5	0.0619 (10)	0.0516 (10)	0.0621 (10)	−0.0085 (8)	0.0234 (8)	−0.0081 (8)
O6	0.114 (2)	0.0975 (19)	0.1034 (18)	−0.0205 (16)	0.0428 (15)	0.0090 (15)
Cu1	0.03799 (16)	0.02756 (15)	0.04243 (16)	−0.00246 (9)	0.02191 (12)	−0.00293 (9)
Na1	0.0476 (5)	0.0339 (4)	0.0542 (5)	0.0006 (3)	0.0243 (4)	−0.0038 (4)
Cl1	0.0566 (4)	0.0601 (4)	0.0712 (4)	0.0090 (3)	0.0184 (3)	0.0195 (3)

### Geometric parameters (Å, °)

**Table:**

C1—C2	1.494 (4)	C18—H18	0.9300
C1—H1A	0.9600	C19—C20	1.402 (3)
C1—H1B	0.9600	C19—H19	0.9300
C1—H1C	0.9600	C20—C21	1.370 (3)
C2—O1	1.433 (2)	C20—H20	0.9300
C2—H2A	0.9700	C21—O4	1.364 (3)
C2—H2B	0.9700	C21—C22	1.428 (3)
C3—C4	1.367 (3)	C22—O3	1.295 (2)
C3—O1	1.371 (2)	C23—O4	1.428 (2)
C3—C8	1.424 (3)	C23—C24	1.496 (3)
C4—C5	1.397 (3)	C23—H23A	0.9700
C4—H4	0.9300	C23—H23B	0.9700
C5—C6	1.360 (3)	C24—H24A	0.9600
C5—H5	0.9300	C24—H24B	0.9600
C6—C7	1.412 (3)	C24—H24C	0.9600
C6—H6	0.9300	C25—O6	1.138 (4)
C7—C8	1.419 (3)	C25—N3	1.353 (5)
C7—C9	1.434 (3)	C25—H25	0.9300
C8—O2	1.298 (2)	C26—N3	1.440 (5)
C9—N1	1.291 (3)	C26—H26A	0.9600
C9—H9	0.9300	C26—H26B	0.9600
C10—C11	1.395 (3)	C26—H26C	0.9600
C10—C15	1.410 (3)	C27—N3	1.384 (6)
C10—N1	1.420 (2)	C27—H27A	0.9600
C11—C12	1.372 (3)	C27—H27B	0.9600
C11—H11	0.9300	C27—H27C	0.9600
C12—C13	1.379 (4)	N1—Cu1	1.9320 (15)
C12—H12	0.9300	N2—Cu1	1.9360 (15)
C13—C14	1.382 (3)	O1—Na1	2.5874 (16)
C13—H13	0.9300	O2—Cu1	1.8907 (13)
C14—C15	1.393 (3)	O2—Na1	2.3247 (15)
C14—H14	0.9300	O3—Cu1	1.8862 (13)
C15—N2	1.414 (3)	O3—Na1	2.3646 (16)
C16—N2	1.297 (3)	O4—Na1	2.5938 (16)
C16—C17	1.432 (3)	O5—Na1	2.4483 (18)
C16—H16	0.9300	O5—H5A	0.8500
C17—C18	1.419 (3)	O5—H5B	0.8499
C17—C22	1.419 (3)	Cu1—Na1	3.3529 (9)
C18—C19	1.355 (3)	Na1—Cl1	2.7726 (10)
			
C2—C1—H1A	109.5	H23A—C23—H23B	108.5
C2—C1—H1B	109.5	C23—C24—H24A	109.5
H1A—C1—H1B	109.5	C23—C24—H24B	109.5
C2—C1—H1C	109.5	H24A—C24—H24B	109.5
H1A—C1—H1C	109.5	C23—C24—H24C	109.5
H1B—C1—H1C	109.5	H24A—C24—H24C	109.5
O1—C2—C1	107.50 (19)	H24B—C24—H24C	109.5
O1—C2—H2A	110.2	O6—C25—N3	128.4 (4)
C1—C2—H2A	110.2	O6—C25—H25	115.8
O1—C2—H2B	110.2	N3—C25—H25	115.8
C1—C2—H2B	110.2	N3—C26—H26A	109.5
H2A—C2—H2B	108.5	N3—C26—H26B	109.5
C4—C3—O1	125.06 (18)	H26A—C26—H26B	109.5
C4—C3—C8	121.16 (18)	N3—C26—H26C	109.5
O1—C3—C8	113.78 (16)	H26A—C26—H26C	109.5
C3—C4—C5	120.48 (19)	H26B—C26—H26C	109.5
C3—C4—H4	119.8	N3—C27—H27A	109.5
C5—C4—H4	119.8	N3—C27—H27B	109.5
C6—C5—C4	120.24 (19)	H27A—C27—H27B	109.5
C6—C5—H5	119.9	N3—C27—H27C	109.5
C4—C5—H5	119.9	H27A—C27—H27C	109.5
C5—C6—C7	120.9 (2)	H27B—C27—H27C	109.5
C5—C6—H6	119.6	C9—N1—C10	123.02 (16)
C7—C6—H6	119.6	C9—N1—Cu1	124.58 (13)
C6—C7—C8	119.65 (18)	C10—N1—Cu1	112.00 (12)
C6—C7—C9	117.51 (18)	C16—N2—C15	123.16 (17)
C8—C7—C9	122.82 (18)	C16—N2—Cu1	124.68 (14)
O2—C8—C7	124.89 (17)	C15—N2—Cu1	112.10 (12)
O2—C8—C3	117.57 (16)	C25—N3—C27	120.8 (4)
C7—C8—C3	117.52 (17)	C25—N3—C26	116.8 (4)
N1—C9—C7	125.74 (18)	C27—N3—C26	122.4 (4)
N1—C9—H9	117.1	C3—O1—C2	117.65 (16)
C7—C9—H9	117.1	C3—O1—Na1	117.35 (11)
C11—C10—C15	119.70 (18)	C2—O1—Na1	124.69 (12)
C11—C10—N1	125.02 (19)	C8—O2—Cu1	126.74 (12)
C15—C10—N1	115.27 (16)	C8—O2—Na1	128.15 (12)
C12—C11—C10	119.8 (2)	Cu1—O2—Na1	104.92 (6)
C12—C11—H11	120.1	C22—O3—Cu1	126.68 (13)
C10—C11—H11	120.1	C22—O3—Na1	129.38 (12)
C11—C12—C13	120.6 (2)	Cu1—O3—Na1	103.57 (6)
C11—C12—H12	119.7	C21—O4—C23	119.18 (16)
C13—C12—H12	119.7	C21—O4—Na1	119.93 (11)
C12—C13—C14	120.9 (2)	C23—O4—Na1	120.61 (13)
C12—C13—H13	119.6	Na1—O5—H5A	115.8
C14—C13—H13	119.6	Na1—O5—H5B	122.1
C13—C14—C15	119.5 (2)	H5A—O5—H5B	107.7
C13—C14—H14	120.2	O3—Cu1—O2	85.28 (6)
C15—C14—H14	120.2	O3—Cu1—N1	178.41 (6)
C14—C15—C10	119.47 (19)	O2—Cu1—N1	94.59 (6)
C14—C15—N2	125.23 (19)	O3—Cu1—N2	94.84 (6)
C10—C15—N2	115.30 (16)	O2—Cu1—N2	179.37 (7)
N2—C16—C17	125.50 (18)	N1—Cu1—N2	85.31 (7)
N2—C16—H16	117.2	O3—Cu1—Na1	43.28 (4)
C17—C16—H16	117.2	O2—Cu1—Na1	42.07 (4)
C18—C17—C22	119.0 (2)	N1—Cu1—Na1	136.65 (5)
C18—C17—C16	118.03 (19)	N2—Cu1—Na1	138.01 (5)
C22—C17—C16	122.98 (18)	O2—Na1—O3	66.12 (5)
C19—C18—C17	121.0 (2)	O2—Na1—O5	103.25 (6)
C19—C18—H18	119.5	O3—Na1—O5	95.10 (6)
C17—C18—H18	119.5	O2—Na1—O1	63.11 (5)
C18—C19—C20	120.9 (2)	O3—Na1—O1	128.73 (5)
C18—C19—H19	119.6	O5—Na1—O1	89.74 (6)
C20—C19—H19	119.6	O2—Na1—O4	127.37 (6)
C21—C20—C19	120.0 (2)	O3—Na1—O4	61.25 (5)
C21—C20—H20	120.0	O5—Na1—O4	81.30 (6)
C19—C20—H20	120.0	O1—Na1—O4	167.51 (6)
O4—C21—C20	126.45 (19)	O2—Na1—Cl1	105.82 (5)
O4—C21—C22	112.61 (16)	O3—Na1—Cl1	111.77 (5)
C20—C21—C22	120.9 (2)	O5—Na1—Cl1	146.43 (5)
O3—C22—C17	125.19 (18)	O1—Na1—Cl1	88.68 (4)
O3—C22—C21	116.57 (18)	O4—Na1—Cl1	93.97 (5)
C17—C22—C21	118.24 (18)	O2—Na1—Cu1	33.02 (3)
O4—C23—C24	107.24 (18)	O3—Na1—Cu1	33.15 (3)
O4—C23—H23A	110.3	O5—Na1—Cu1	102.27 (4)
C24—C23—H23A	110.3	O1—Na1—Cu1	96.02 (4)
O4—C23—H23B	110.3	O4—Na1—Cu1	94.39 (4)
C24—C23—H23B	110.3	Cl1—Na1—Cu1	111.24 (3)
